# Impact of digital automation of institutional pathways on test-ordering patterns in a primary health care

**DOI:** 10.31744/einstein_journal/2026AO2184

**Published:** 2026-09-09

**Authors:** Anderson Aires Eduardo, Lídia Maria Lourençön, Adriana Morelli Martins, Roberto Alves de Sousa

**Affiliations:** 1 Hospital Israelita Albert Einstein Digital Platform Department São Paulo SP Brazil Digital Platform Department, Hospital Israelita Albert Einstein, São Paulo, SP, Brazil.

**Keywords:** Decision support systems, Clinical data, Electronic medical records, Obesity, Evidence-based guidelines, Digital automation

## Abstract

**Objective:**

To evaluate the impact of digital automation of institutional pathways on the adherence of test ordering to evidence-based best practices in a primary care setting for obesity.

**Methods:**

Observational, single-center, retrospective study comparing the periods before (2023) and after (2024) the implantation of a clinical decision support system. Outcomes: volume of tests adherent to protocols, distribution of ordering mix (entropy/perplexity), and test-specific differences (Poisson rate ratios). Rates per 100 visits were estimated, and incidence rate ratios (IRR) with 95% confidence intervals (95%CI) were estimated. The Mann-Whitney test was used to compare adherent tests per visit, alongside descriptive analyses (top-10 both in absolute and percentage changes).

**Results:**

Total orders: 347 (before) *versus* 787 (after); distinct test types: 117 *versus* 120. Adherent tests: 179 *versus* 413; IRR=1.63 (95%CI=1.37-1.96; p<0.001). Median of adherent tests per visit: 1.0 (before) *versus* 3.0 (after) (p=0.027). Perplexity: 70.9 (before) *versus* 57.6 (after), indicating increased concentration of orders in protocol-based tests. Among the top-10 absolute growth tests, 9/10 were protocol-based; among the top-10 by percentage change, 0/10.

**Conclusion:**

Digital automation of the pathway was associated with greater adherence to institutional protocols and with a test-ordering pattern more concentrated in core protocol tests.

## In Brief

Digital automation of an institutional obesity pathway via a clinical decision support system significantly increased adherence to evidence-based test ordering in primary care, reduced variability and concentrated orders on core protocol-based tests, indicating improved standardization of clinical practice.

## Highlights

Clinical decision support systems increased protocoladherent test ordering (IRR=1.63).

Median adherent tests per visit rose from 1.0 to 3.0.

Ordering became more concentrated (perplexity ↓ 70.9 → 57.6).

Gains driven by core protocol tests, not by low-frequency tests.

## Graphical abstract

**Figure:**
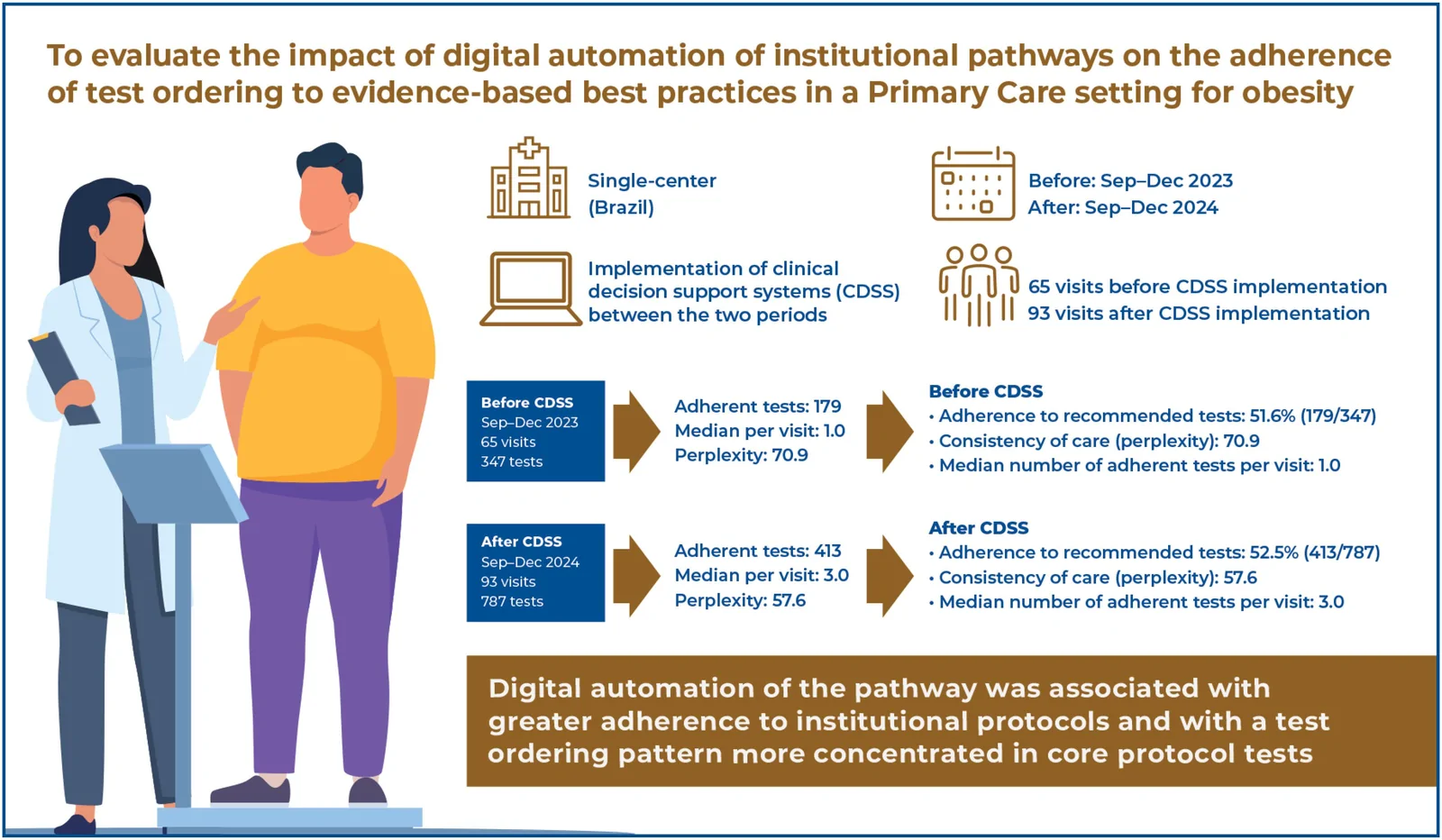

## INTRODUCTION

Adherence of healthcare professionals to institutional protocols of best medical practices is essential to ensure quality of care, patient safety, and operational efficiency in healthcare institutions.^(1-3)^ However, challenges such as protocol complexity, workload burden, and variability in clinical practice can compromise the consistent implementation of these guidelines.^(4,5)^ Particularly when addressing multifactorial conditions such as obesity, the difficulty in aligning clinical conduct with institutional recommendations may lead to failures in clinical management and inappropriate test ordering, thus affecting care effectiveness and healthcare costs.^(6,7)^ In this context, clinical decision support systems (CDSS) emerge as promising technological tools to assist in the application of evidence-based recommendations at the point of care.^(8)^

Clinical decision support systems are platforms that integrate patient clinical data with institutional protocols and scientific evidence, providing professionals with relevant information for real-time decision-making. Studies indicate that the use of these systems can reduce medical errors, standardize clinical conduct, and improve adherence to clinical guidelines.(9,10) Moreover, their implantation fosters a collaborative and digital environment, promoting continuous improvement in medical practice.^(9,11)^

In primary and vulnerable care contexts, digital systems based on CDSS have significantly enhanced service delivery, increasing clinical independence of professionals and adherence to care standards even in resource-limited settings.^(12)^ However, to ensure acceptance and sustained use, it is critical that CDSS fit appropriately into clinical workflows, are perceived as useful, and have ongoing user support.^(13)^

## OBJECTIVE

This study aimed to empirically evaluate the impact of pathway automation (through a clinical decision support systems) on healthcare professionals’ adherence to institutional guidelines for appropriate test ordering in obesity-related visits. We analyzed changes in test-ordering patterns before and after the implementation of a clinical decision support systems that automated the institutional protocol for obesity (Obesity Pathway - publicly available at: https://medicalsuite.einstein.br/Paginas/Home.aspx), seeking to understand the extent to which this tool may contribute to a clinical practice more closely aligned with best available evidence-based medical practices and institutional objectives, focusing on the Primary Health Care services.

## METHODS

### Study design

This was a single-center, observational, exploratory, and retrospective study conducted using a pre-existing institutional database from the electronic medical record (EMR) (Cockpit Clínicas™) routinely used by healthcare professionals at Einstein Clinics. The CDSS was implemented (properly embedded) in Cockpit Clínicas™ in the first quarter of 2024. No specific staff training was required as the CDSS recommendations were integrated into the EMRs routinely used by the professionals (for detailed description, please refer to Eduardo et al. 2026).^(14)^ Therefore, data from two groups of visits were compared: before (group 1) and after (group 2) the implantation of the system, considering the same time frame and season of the year for each group (September-December 2023 and September-December 2024, respectively). Only data from patients attending a first medical consultation with obesity as the reason for visit (ICD-10 E66) and for whom clinical tests were ordered were included in the analysis.

### Data

The data analyzed consisted of lists of medical tests ordered for patients, as recorded in the institutional EMR system. The study population comprised individuals who spontaneously sought care at the Einstein Clinics within the time periods defined in the project (see Study Design section, above), without any intervention from the researchers. The primary unit of comparison in the study was the outpatient medical visits. The tests ordered in each visit were classified as adherent or non-adherent to the institutional protocol based on a predefined list of test names recommended in that document. Thus, a tabular dataset was obtained with the following columns: "ID" (an integer artificially generated to uniquely identify each medical visit); "exam_name" (text column containing the names of the tests); "exam_code" (text column containing the identification code of the test in the outpatient EMR system); "pathway_check" ("True" or "False," indicating whether the ordered test was included in the institutional protocol or not).

### Outcomes and metrics

The metrics used in this study were: (i) descriptive statistics (e.g., mean, median, standard deviation [SD], coefficient of variation, etc.) for the total number of tests per year and for the total number of distinct test types; (ii) descriptive statistics for the total number of adherent tests per year; (iii) rate of adherent tests per 100 visits and distribution per visit; (iv) difference between group 1 (2023) and group 2 (2024) for each test, with incidence rate ratio (IRR); (v) analysis of ordering mix concentration using Shannon entropy and perplexity - entropy is defined as H=−∑pi log pi , where *pi* represents the proportion of each test type, while perplexity is computed as *e*^H^, representing the effective number of equally probable test types;^(15,16)^ (vi) descriptive comparison between the top-10 tests by absolute growth and the top-10 by percentage growth.

### Statistical analyses

For the after/before rate ratio (i.e., group 2/group 1, or 2024/2023), a count model was employed assuming a Poisson process with exposure equal to the number of visits in the analyzed periods (with Haldane-Anscombe correction when necessary), estimating IRR, 95% confidence interval (95%CI), and two-tailed p-values. The distribution of adherent tests per visit was compared between groups using the Mann-Whitney test. We also calculated the raw proportions of adherent tests among all tests ordered in each period, as a supplementary descriptive analysis (i.e., the hypothetical scenario assuming each test order in the sample is independent).

Furthermore, following the approach of other authors^(15,16)^ we also calculated data entropy (employing log base 2) for each group based on the frequency distribution of adherent tests per visit in each period. From the entropy, we estimated the perplexity metric, which allowed us to approximate the effective number of tests for each analyzed period (i.e., before vs. after). This metric helped us to further interpret the change in test-ordering patterns before and after CDSS implantation.

All statistical procedures were conducted in a Python programming environment (v3.11), employing conventional data science libraries (e.g., pandas, numpy, math, matplotlib). The analysis script is available in the dedicated GitHub repository: https://github.com/AndersonEduardo/digital-pathways-improves-healthcare

### Ethical considerations

All data were collected and anonymized within the private institutional environment by the database administrator of Cockpit Clínicas™. The study, being retrospective in nature, involved no contact with patients or their physicians, nor any access to sensitive information, and rigorously followed all ethical standards established institutionally and by current legislation. Approval by the *Hospital Israelita Albert Einstein* ethics committee was granted under registration number CAAE: 87453325.9.0000.0071; #7.676.517.

## RESULTS

The sample comprised 65 visits in 2023 (before) and 93 visits in 2024 (after CDSS implantation). The mean patient age was 39.5 (±14.5) in the before and 41.0 (±13.1) in the after groups. The 2023 sample comprised 64.6% women (n=42) and 35.4% men (n=23). Regarding the professionals involved, 29 physicians were identified in the 2023 sample (75.5% women and 24.5% men), and 40 in the 2024 sample (70% women and 30% men). The total number of medical test orders was 347 and 787, respectively in 2023 and 2024, and the number of distinct test types was 117 and 120, respectively. A marked increase in the overall volume of orders was observed between the years (∼127%). We also found that the volume of low-frequency tests (i.e., n<3) dropped from 85 (∼24.5% of requests in 2023) to 66 test types (∼8.4% of requests in 2024).

Regarding adherence, the total number of protocol-adherent tests increased from 179 to 413 (∼131%). When normalized by clinical exposure (i.e., visits in the period), the rate increased significantly: IRR=1.63 (95%CI=1.37-1.96; p<0.001). This indicates a significant rise in the adoption of protocol-based tests (∼134%). When examining the raw proportions, we found that 51.56% (179/347) were adherent in 2023 and 52.5% (413/787) in 2024. This suggests that part of the observed increase in adherent tests accompanied the increase in overall test ordering volume.

The distribution of adherent tests per visit showed an increase in the median from 1.0 in 2023 (before) to 3.0 in 2024 (after) (interquartile range [IQR]=0-5 *versus* 0-9; Mann-Whitney p=0.027). The mean (±SD) per visit also increased (2.75±3.59 and 4.44±4.73, respectively). Notably, adherence variability (measured by the coefficient of variation), despite the increase in the visits and test-order volumes, decreased after CDSS implantation: from 1.30 to 1.07 (-21.5%).

The diversity of the test mix (Shannon entropy) was 6.15 bits in 2023 and 5.85 bits in 2024. The corresponding perplexity decreased from 70.9 to 57.6 for effective tests, even with the observed increase in visits and orders. Together, these metrics indicate a higher concentration of orders within a smaller effective set of tests (i.e., after, compared to before), consistent with the expected standardization following automation of the institutional pathway.

In the test-level analysis, the top-10 tests with the greatest absolute growth included 9/10 protocol-based tests, while the top-10 by percentage change included 0/10 protocol-based tests. This pattern indicates that absolute gains were concentrated in core protocol tests, whereas large percentage changes occurred in low-base items that were mostly non-protocol tests (likely requested in specific clinical investigations).

Tables 1 and 2 show the top-10 by absolute and relative values, respectively. The table presents the annual counts for each test in 2023 and 2024, absolute and percentage changes (Δ and Δ%), incidence rate ratios (IRR), 95%CI, and p-values. Tests are ordered according to the complete dataset available from: https://github.com/AndersonEduardo/digital-pathways-improves-healthcare/blob/main/outputs/output_completo_normalizado_com_significancia.xlsx The overall results remained consistent in robustness analyses with normalization per 100 visits and stability filters (e.g., tests with zero counts).

**Table 1:** Top-10 tests according to the absolute change in requests between the pre-implantation period (2023) and the post-CDSS implantation period (2024)

Rank	Test name	Protocol-based?	Before	After	Δ absolute	Δ %	IRR (after/before)	95%CI	p value
1	TSH - Thyroid-Stimulating Hormone	Yes	12	39	27	225.00	2.27	1.19-4.34	0.01*
2	Complete Blood Count with Platelets	Yes	22	47	25	113.64	1.49	0.90-2.48	0.12
3	Glycated Hemoglobin - Blood (EDTA)	Yes	10	33	23	230.00	2.31	1.14-4.68	0.02*
4	Fasting Glucose	Yes	20	43	23	115.00	1.50	0.88-2.55	0.13
5	Total Cholesterol and Fractions - Serum	Yes	19	38	19	100.00	1.40	0.81-2.42	0.23
6	Creatinine	Yes	10	29	19	190.00	2.03	0.99-4.16	0.05
7	Triglycerides	Yes	16	34	18	112.50	1.49	0.82-2.69	0.19
8	AST (SGOT) - Aspartate Aminotransferase	Yes	7	25	18	257.14	2.50	1.08-5.77	0.03*
9	ALT (SGPT) - Alanine Aminotransferase	Yes	7	25	18	257.14	2.50	1.08-5.77	0.03*
10	HCV Serology	No	1	18	17	1700.00	12.58	1.68-94.24	0.01*

The number of requests before and after the implantation, the absolute and percentage variation, the incidence rate ratio (IRR) with the corresponding 95% confidence interval (95%CI), and the p-values obtained from the rate ratio test.

Tests classified as "Protocol-based" correspond to those recommended in the institutional pathways; the only non-protocol test included among the top ten was HCV serology. p< 0.05 indicate statistically significant differences (values marked with asterisk).

**Table 2:** Top-10 tests according to the percentual change in requests between the pre-implantation period (2023) and the post-CDSS implantation period (2024)

Rank	Test name	Protocol-based?	Before	After	Δ absolute	Δ %	IRR (after/before)	95% CI	p value
1	HCV Serology	No	1	18	17	1700.00	12.58	1.68-94.24	0.01
2	Hepatitis B - Anti-HBs	No	1	16	15	1500.00	11.18	1.48-84.33	0.02
3	Echocardiogram with Doppler	No	1	8	7	700.00	5.59	0.70-44.71	0.10
4	Fecal Occult Blood Test - Stool without Preservative	No	1	8	7	700.00	5.59	0.70-44.71	0.10
5	1,25-Dihydroxy Vitamin D, Quantification	No	0	6	6	600.00	8.39	0.47-150.17	0.15
6	Colonoscopy with Home Preparation and Anesthesia	No	0	6	6	600.00	8.39	0.47-150.17	0.15
7	Pelvic and Transvaginal Ultrasound	No	2	14	12	600.00	4.89	1.11-21.53	0.04
8	Real-Time PCR for Coronavirus COVID-19 - Swab	No	1	7	6	600.00	4.89	0.60-39.77	0.14
9	VDRL (Venereal Disease Research Laboratory Test)	No	3	17	14	466.67	3.96	1.16-13.51	0.03
10	Prolactin	No	0	4	4	400.00	5.59	0.30-105.77	0.25

The table shows the number of requests before and after CDSS, the absolute and relative variation (Δ%), the incidence rate ratio (IRR) with 95% confidence interval (95% CI), and the corresponding p-values.

Unlike the ranking by absolute growth (see Table 1), all tests listed in this analysis were non-protocol, with emphasis on HCV serology and hepatitis B serology (anti-HBs), which showed the greatest percentage changes.

p<0.05 indicate statistically significant differences (values marked with asterisk).

## DISCUSSION

Overall, the findings provide empirical evidence that automation of an institutional pathway (focused here on obesity) through the implantation of a CDSS was associated with increased adherence to test-ordering recommendations in Primary Health Care (PHC). At the visit level, the total number of adherent tests increased by more than 130% between 2023 and 2024, with an increase in the median number of tests per visit (from 1.0 to 3.0) and a reduction in relative variability. These results suggest that the introduction of the CDSS helped align clinical practice with institutional guidelines and reduce heterogeneity among physicians.

The entropy and perplexity analyses support this pattern. Despite the observed increase in the volume and variety of ordered tests, perplexity decreased (from 70.9 to 57.6 effective tests), indicating greater concentration of orders within the set of tests recommended by the institutional pathway. This metric, often used in biological sciences and information science to estimate effective diversity of categories (or entities),^(15,16)^ proved to be useful in the clinical context. The observed drop in perplexity reflects that professionals shifted away from ordering "rare" tests and focused on the core pathway tests. This finding is consistent with the hypothesis that digital automation of evidence-based institutional protocols fosters consistency of care in PHC settings.

Considering the ranking analysis, the top-10 by absolute growth included nine protocol-based tests, demonstrating that the most relevant clinical gain was concentrated in the core components of the pathway examined in this study. By contrast, the top-10 by percentage change consisted mostly of non-protocol, low-frequency tests, reflecting occasional fluctuations that are institutionally less relevant. This contrast illustrates the importance of complementary analyses to avoid biased interpretations driven by small-base effects (i.e., tests with low counting).

Our results are in line with the literature on CDSS. Systematic reviews indicate that such systems, when well-integrated into operational workflows, can increase guideline adherence and reduce clinical variation.^(9,17)^ Evidence specifically on order sets and laboratory pathways shows improvements in consistency and rationalization of test ordering.^(18,19)^ Recent studies on behavior-oriented interventions in EMRs, such as "nudges," also suggest gains in adherence without increasing cognitive load.^(20,21)^ Within this context, our findings contribute by showing that, in primary care and in a complex condition such as obesity, digital automation of pathways yields measurable standardization outcomes.

From a clinical-operational perspective, greater adherence to protocols has positive implications. Standardization may reduce waste, improve allocation of laboratory resources, and enhance patient safety by minimizing inappropriate orders. Moreover, it promotes data comparability, which is fundamental for quality monitoring and outcome assessment. Contrarily, reduced diversity of orders may raise concerns about the risk of "over-standardization," that is, the potential risk of suppressing individualized clinical investigations demanded by complex patient cases, especially in PHC. Therefore, the use of CDSS in the automation of institutional best-practice protocols (such as evidence-based guidelines) should be implemented with a broad physician involvement, accompanied by pertinent EMR flexibility, and subject to periodic protocol reviews.

The study is not without its limitations. It was retrospective, single-center, and limited to first consultations for obesity, which inherently restricts the generalizability of the findings to other contexts. In addition, it was not possible to control for confounding factors such as patients’ sociodemographic profile and physicians’ professional experience (although institutional leadership reported no major changes at Einstein Clinics during the study periods). Also, while the statistical analysis employed robust methods (Mann-Whitney test for skewed distributions, Poisson models for rates, entropy and perplexity for the effective number of tests ordered), longitudinal analyses with mixed models may provide greater granularity.

In summary, the results indicate that digital automation of institutional pathways via CDSS significantly increased adherence to evidence-based recommendations and promoted standardization in test ordering in a PHC context. The combined use of traditional metrics (e.g., rates, IRR) and diversity metrics (e.g., entropy, perplexity) enabled both detection and a richer interpretation of changes in test-ordering patterns. Future multicenter, prospective studies with assessment of clinical and economic outcomes are needed to confirm and expand these findings.

## CONCLUSION

The digital automation of institutional pathways through a clinical decision support systems significantly increased adherence to guideline-based laboratory test ordering in primary care for patients with obesity. Beyond higher rates of protocol-concordant requests, the system promoted greater consistency and reduced variability in physicians’ ordering behavior, as evidenced by decreased entropy and perplexity of test distributions. These findings highlight the potential of digital pathways to translate evidence into routine care and strengthen institutional governance over clinical processes. Future studies should employ multicenter, prospective designs to evaluate clinical and economic outcomes in a broader context to further explore and validate our findings.

## Data Availability

The underlying content is contained within the manuscript.
